# The Advent of a New Era in Digital Healthcare: A Role for 3D Printing Technologies in Drug Manufacturing?

**DOI:** 10.3390/pharmaceutics14030609

**Published:** 2022-03-10

**Authors:** Ioannis I. Andreadis, Christos I. Gioumouxouzis, Georgios K. Eleftheriadis, Dimitrios G. Fatouros

**Affiliations:** Laboratory of Pharmaceutical Technology, Department of Pharmacy, Aristotle University of Thessaloniki, 54124 Thessaloniki, Greece; andreadi@pharm.auth.gr (I.I.A.); gioumouxo@pharm.auth.gr (C.I.G.); dfatouro@pharm.auth.gr (D.G.F.)

**Keywords:** 3D printing, pharmaceutical product, Industry 4.0, personalized medications, drug delivery systems, digital healthcare

## Abstract

The technological revolution has physically affected all manufacturing domains, at the gateway of the fourth industrial revolution. Three-dimensional (3D) printing has already shown its potential in this new reality, exhibiting remarkable applications in the production of drug delivery systems. As part of this concept, personalization of the dosage form by means of individualized drug dose or improved formulation functionalities has concentrated global research efforts. Beyond the manufacturing level, significant parameters must be considered to promote the real-time manufacturing of pharmaceutical products in distributed areas. The majority of current research activities is focused on formulating 3D-printed drug delivery systems while showcasing different scenarios of installing 3D printers in patients’ houses, hospitals, and community pharmacies, as well as in pharmaceutical industries. Such research presents an array of parameters that must be considered to integrate 3D printing in a future healthcare system, with special focus on regulatory issues, drug shortages, quality assurance of the product, and acceptability of these scenarios by healthcare professionals and public parties. The objective of this review is to critically present the spectrum of possible scenarios of 3D printing implementation in future healthcare and to discuss the inevitable issues that must be addressed.

## 1. Introduction

We are truly living a technological revolution in the manufacturing of finished goods, in view of the historic gateway of the fourth industrial revolution. In this concept, the major impact of the fourth industrial revolution, also known as Industry 4.0, is based on overriding the fundamental limitations in the interface between humans and machines [1]. In a continuation of current manufacturing strategies, automation-promoting digital technologies, e.g., artificial intelligence (AI) and the Internet of Things (IoT), will be utilized in an array of public and private domains to reveal personalized approaches for any given individual [2]. As part of Industry 4.0, three-dimensional (3D) printing is expected to have a vital role in the manufacturing and mass customization of complex and highly personalized products [3,4].

**Figure 1:**
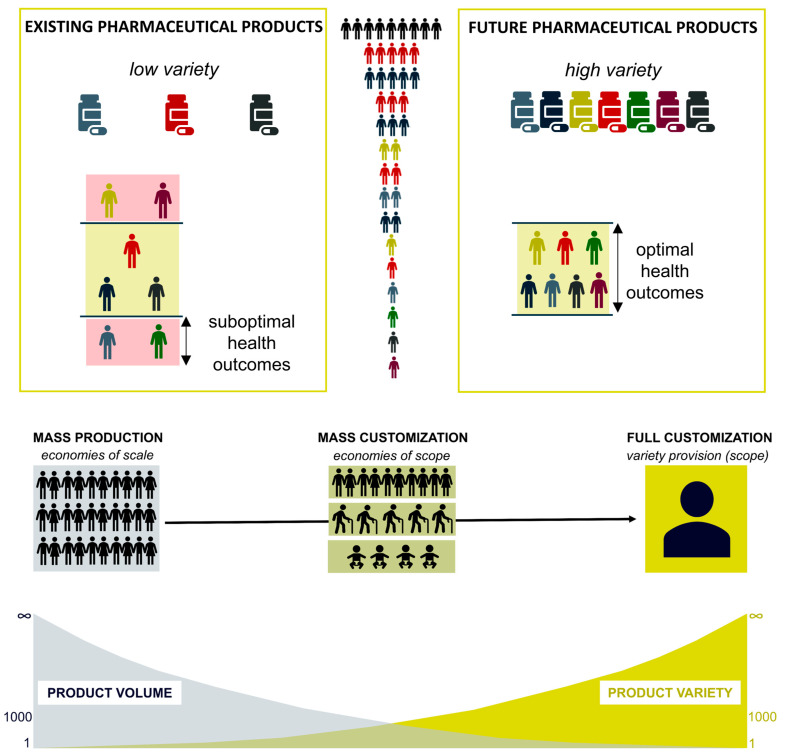
The need for personalization of pharmaceutical products and changes in product volume and va-riety due to product customization according to the needs of individual patients. This figure has been reproduced with permission from © 2021 Rydvikha Govender [6].

Among the different fields that have benefitted from 3D printing technology, considerable research activity has been focused on the manufacturing of 3D-printed pharmaceuticals. The personalization of drug delivery systems is apparent in future digital healthcare by revolutionizing existing and well established pharmaceutical manufacturing techniques [5]. The implementation of such scenarios is principally based on the establishment of interactive feedback between the needs of each patient and the pharmaceutical product (Figure 1) [6]. Therefore, 3D printing has already shown its great potential in the development of customized drug products by means of product shape/size and drug dose or the attribution of special functionalities, e.g., controlled release and advanced mucoadhesive or drug permeability properties [7,8].

Although the regulatory framework and the clinical translation of 3D-printed drug products is still in its infancy [9,10], different scenarios have already been proposed in the literature, considering the integration of this technology in future healthcare settings. In the beginning, this review article will provide an overview of 3D-printed drug delivery systems, considering the potential of this technology for dose personalization, controlled drug release, response to polypharmacy, and special patient populations. Next, we provide an overview of the proposed scenarios and general challenges for installation of 3D printers at different manufacturing sites, i.e., industry, community and hospital pharmacies, and patients’ houses. Finally, we provide a critical discussion on the implementation of these scenarios related to existing drug shortages in hospitals, the need for an advanced networking capacity between different manufacturing sites, and the requirement for establishment of a profound regulatory framework.

## 2. Application of 3D Printing in Drug Delivery

### 2.1. Personalization of Drug Dose

The response of each patient to a specific treatment is not always identical, as it may vary according to the patient’s age, biomarkers, and genetic characteristics [11]. Novel methods (e.g., DNA sequencing and proteomics) have proven the connection between an individual’s biologic characteristics and the progress of a disease or the successful treatment thereof [12]. Precision medicine aims to tailor a specific treatment for each individual based on their biologic characteristics, along with other socioeconomic parameters and personal preferences [11]. Use of the term of personalized medicine increased in the early 2000s due to the first human genome sequencing and genomic data collection that led to treatment guidelines based on the prediction of drug efficiency for individuals with specific genetic variations. Although “next-generation” sequencing methods and biobanks of human DNA specimens are becoming widely available, the direct implementation of this information in clinical practice is still hindered by the absence of appropriate tools and reasons concerning costs and staff training [13]. 3D printing can be utilized as a tool for drug manufacturing, with individualized doses depending on the patient’s characteristics by scaling of the physical size and digital design of the dosage form [14]. With additive manufacturing, the issues of inaccurate dosing and dose variance from cutting tablets can be addressed, and a greater range of doses can be precisely produced [14,15]. Pietrzak et al. employed 3D printing to manufacture tablets containing theophylline with varying drug doses, ranging from 60 mg to 300 mg, and tested the dose accuracy while digitally regulating the desired drug strength [16]. Zheng et al. compared 3D-printed and manually subdivided tablets containing spironolactone or hydrochlorothiazide in terms of accuracy of mass and drug dose and showcased the compliance of the 3D-printed tablets with the European and Chinese Pharmacopoeia standards. The drug dose was adjusted by altering the diameter and height of the tablet, as the dose was found to be linearly related to the volume. The prepared formulations were also administered as personalized regimens to patients of a Grade III-A hospital, with high rates of acceptance by both patients and healthcare professionals [17].

### 2.2. Regulation of Drug Release

Another way to personalize a treatment according to an individual’s needs is to modify and regulate the drug release profile of the formulation. 3D printing can be utilized to produce dosage forms with varying release profiles, e.g., immediate, sustained, or pulsatile drug release [15], by changing the geometry, the infill, or the selected polymers of the 3D-printed formulation [18]. Kadry et al. produced 3D-printed tablets containing hydroxypropyl-methylcellulose (HPMC) and diltiazem and tested the effects of the inner structure, well known as infill percentage, and design patterns on the drug release performance. The researchers managed to manufacture tablets with a variety of release profiles (immediate, sustained, delayed, and pulsatile) and proved that the pharmacokinetic profiles of these tablets when administered in vivo in rats were in accordance with the in vitro release studies [19]. Gorkem Buyukgoz et al. also investigated the effect of the design of 3D-printed tablets on drug release profiles by altering the size of the tablet, the drug loading of the feedstock (polymeric filament), and the special accumulation of the drug in the tablet. The in vitro release profiles of the various tablets, as well as the release kinetics in each case, were explored to define the mechanism of drug release. The importance of the surface area-to-volume ratio of the 3D-printed tablets for the prediction of the release profile was emphasized, and regulating the dose of the 3D-printed tablets while keeping the release rate constant was proposed [20]. Wen et al. used 3D printing to combine gastro-retention and controlled drug release in a single tablet and achieved zero-order drug release for 10–12 h by simply changing the internal structure of the tablet [21]. Gioumouxouzis et al. developed a 3D-printed pH-responsive oral tablet for the controlled delivery of 5-fluorouracil to the colon (pH 7.4) [22], as well as a 3D-printed osmotic tablet that can modify the release of drugs based on the design of the dosage form [23]. The release profile can be tailored through 3D printing, not only for orally administered dosage forms, but also for other dosage forms, e.g., personalized suppositories [24].

### 2.3. Personalized Treatment for Geriatric Patients and Polypharmacy

**Figure 2:**
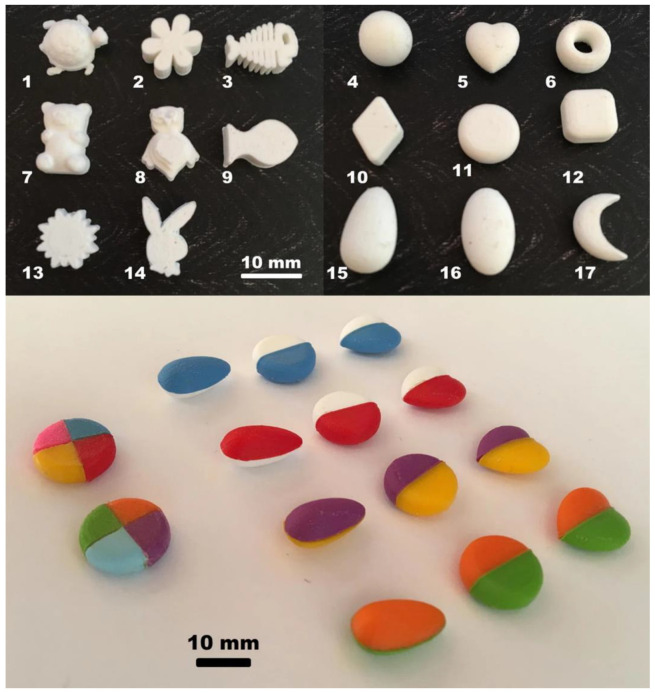
3D-printed solid dosage forms in various shapes (**upper part**) and 3D-printed polypills (**lower part**) [33].

A special group of patients attracting attention in the field of medicine is the geriatric population; older people are usually affected by more than one medical condition. The use of various drugs, the variance of response to pharmacotherapy, swallowing difficulties, and the inability to handle medication (e.g., pill cutting) all lead to the need for personalized medicine for this group [25]. Polypharmacy is a problem connected with the prescription of multiple drugs for a single patient, as well as an issue that must be addressed, considering that it usually leads to reduced patient compliance with therapy and higher possibility of adverse reactions and drug interactions [26,27]. 3D printing offers a possible solution to these problems by introducing the concept of polypills: a single tablet containing multiple drugs, customized for a specific patient according to the therapeutic needs [15]. The combination of multiple drugs in 3D-printed polypills has been approached by many research groups. Khaled et al. created a 3D-printed polypill combining three drugs in separate compartments with controlled release rates and possible benefits for treating hypertensive diabetics [28]. Other research groups developed polypills via 3D printing, comprising four or five different drugs commonly prescribed for cardiovascular diseases [29,30], while Robles-Martinez et al. worked on a multilayered 3D-printed polypill containing six different drugs [31]. Polypills have also been proposed as a means for personalized supplementation [32]. Fastø et al. studied how polypharmacy patients comprehend 3D-printed tablets and what their preferences are concerning the shape, color, and design of their medication (Figure 2). Most patients prefer shapes similar to conventional tablets, whereas different colors and designs were chosen by each individual based on their personal taste. Polypills were found to be a generally accepted concept by polypharmacy patients due to the minimization of the number of tablets they need to consume within a day [33]. Apart from the shape of the 3D-printed tablet, patient-driven sensory evaluation is further described by the swallowability of the dosage forms. A recent study showed that 3D-printed dosage forms with rough edges were hard to swallow, depending on the shape (e.g., pyramid and cuboctahedron) [34]. The process-driven texture, i.e., surface roughness, has also been reported to be higher for 3D-printed tablets compared to conventional tablets, leading to swallowing difficulties [35,36]. Hence, further studies on the control of surface texture during and after 3D printing are required in order to increase patient acceptability.

### 2.4. Personalized Treatment for Pediatric Patients

Pediatric patients are a group of great importance in the field of pharmacotherapy, since special attention must be given to the safety and efficiency of their treatment. Dosage forms aimed for administration in children are less readily available and should meet very specific criteria in terms of dosing, toxicity, and organoleptic characteristics. The dose must be regulated very precisely and must be decided based on the child’s age, developmental stage, and bodily characteristics. All ingredients used, including active substances and excipients, should be extensively studied for their possible toxicity in children of all ages. Finally, ease of administration and taste are major concerns for the pediatric population, especially taste, since children express greater sensitivity to bitter ingredients in comparison to adults [37]. The most common pediatric formulations are administered *per os* and mainly include solutions, suspensions, and orodispersible films, powders and tablets (including small, scored, orodispersible, chewable, or mini tablets) [38]. The use of 3D printing in the preparation of personalized pediatric formulations is a developing field, where healthcare professionals or patients themselves can choose the shape and color of the 3D-printed medication in a safe and efficient way [39]. Furthermore, as swallowing difficulty is a major problem for young children, 3D printing is a suitable technology for the manufacturing of orodispersible films and tablets and chewable dosage forms [15]. 

**Figure 3:**
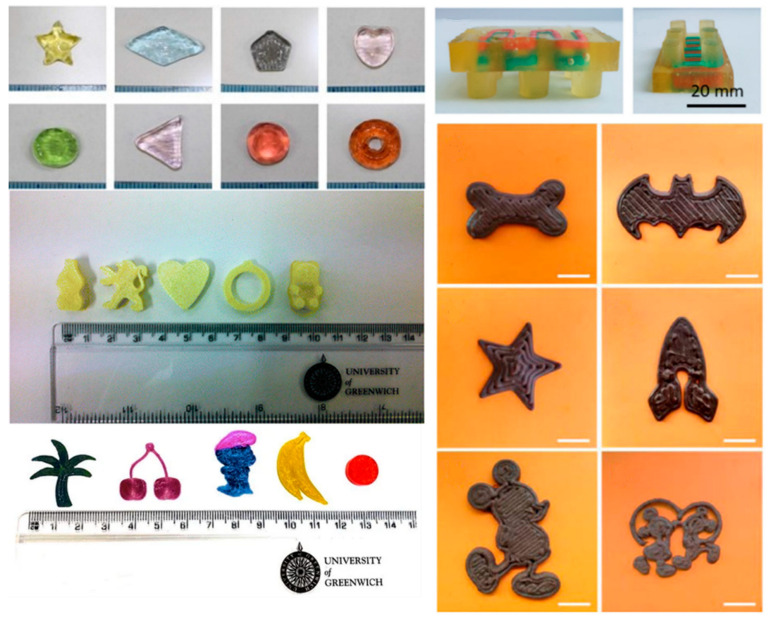
3D-printed solid dosage forms for pediatric patients; gummy dosage forms (**upper left**) [41]; dosage forms shaped like candy (Starmix^®^) (**middle left**) [42]; taste-masked chewable dosage forms in various shapes (**lower left**) [43]; soft chewable Lego ™-shaped dosage form (**upper right**) [44]; chocolate-based dosage forms in various shapes (**lower right**) [40].

Figure 3 presents representative examples of 3D-printed formulations that can be administered to pediatric patients. Karavasili et al. 3D-printed dosage forms based on chocolate for the administration of both hydrophilic and lipophilic drugs to children. Ibuprofen and paracetamol were chosen as the incorporated drugs and the chewable chocolate formulations were printed in designs of simple shapes or popular cartoon characters so that the children could choose their favorite character and be a part of the process [40]. Another group used 3D printing to develop soft chewable pediatric-friendly drug-loaded gummies consisting of gelatin and HPMC in various shapes and colors [41], while Scoutaris et al. [42] and Tabriz et al. [43] improved the palatability of bitter drugs by incorporation in 3D-printed chewable tablets in the form of candies. Soft chewable dosage forms based on gelatin in the form of Lego™ bricks were also fabricated using a novel embedded 3D printing technique [44]. Other attempts have been made for the application of 3D printing in the development of other common pediatric dosage forms, including mini-caplets [45], mini-tablets [46], and orodispersible tablets [47], with the capability of dose regulation according to the patient’s needs. Cui et al. also focused on dosing regulation through 3D printing for the manufacturing of tablets for pediatric patients by developing a novel drop-on-powder technology. This method proved to be more accurate compared to the traditional tablet-cutting methods, while the drug release profile was not affected [48].

A study on the preferences of children aged 4 to 11 concerning the visual representation of 3D-printed tablets was conducted by Januskaite et al. [49]. Four different 3D printing technologies were compared, and the initial results showed the preferred 3D printing technique, although most of the participants changed their opinion after being informed that one of the other formulations was chewable, proving that chewable tablets are indeed favored by pediatric patients [49]. Healthcare professionals have an overall positive attitude towards 3D-printed oral formulations for pediatric patients, emphasizing the benefit of precision and personalization of doses and the production of polypills in cases of polypharmacy. However, some concerns were raised, mainly related to the size of the oral formulation, the dose-verification process, and the total time required for the manufacturing of the dosage forms [50]. 

### 2.5. Personalized Treatment for Visually Impaired Patients

**Figure 4:**
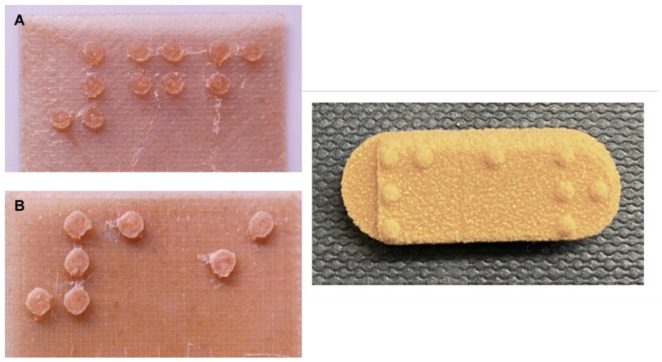
3D-printed oral films (**left**) and tablet (**right**) containing Braille characters for identification of the dosage form by visually impaired patients. The respective diameters of the Braille patterns are 1.6 mm (**A**), 2 mm (**B**), and 1.5 mm (**right**) [51,52].

Visually impaired patients may encounter problems when receiving medications, such as difficulty reading labels and differentiation of drugs, especially after their removal from or the deformation of the packaging. With 3D printing, the opportunity to print identifying characters on the tablets themselves arises (Figure 4). A first attempt was shown by printing Braille and Moon characters onto orally disintegrating tablets. These characters could refer to drug indications, dose, or other information. A visually impaired volunteer also verified the readability of the characters [51]. Intraoral films with incorporated Braille characters were also 3D-printed for the personalized treatment of visually impaired patients, and an in vivo haptic evaluation study was conducted by recruited volunteers, who confirmed the readability of the embedded text and confirmed the potential of 3D printing for the personalization of their medication [52]. 

## 3. Future Settings of 3D-Printed Pharmaceuticals and Challenges

The discussion around the setting of 3D printing of drugs in the future has already started, with a main focus on the advantages and challenges of each possible setting. Such settings for the installation of 3D printers include in the patient’s house, in the pharmaceutical industry, in the community pharmacy, or in the hospital pharmacy. Beer et al. published a detailed case study on the different theoretical scenarios mentioned here, where participants from various backgrounds related to the healthcare system were interviewed, sharing their perspective on the future of 3D printing as a manufacturing method for personalized treatments [53].

### 3.1. Patient’s House

As 3D printers are becoming cheaper and more easily accessible to the public, they could be used as home printers for the production of one’s own medicine [54]. Printing a patient’s medicine in their own house is widely discussed and is quite popular as a scenario. However, the suitability of this approach for the production of drugs has been questioned by many specialists, as the quality of the final product is not guaranteed. This is especially worrying for pediatric dosage forms, where higher precision and safety should be achieved in all cases [55]. Additionally, another problem mentioned is the possible unintentional or intentional misuse of the home-produced drugs if no control is instituted [53,56]. Although involving patients in the process of their treatment has been proven to be beneficial for the outcome of treatment, meticulous training of the patients or the person taking care of them on the printing technology and assessment of the final product seems impractical [14]. Nevertheless, there is the possibility of remote control of the printing process by specialists and medical staff [56], as there is always the chance that patients do not show an interest in participating in their treatment plan [54]. However, this setting is still considered the least realistic by healthcare professionals [53].

### 3.2. Pharmaceutical Industry

Despite the technological advances and extensive research on personalized medicine, current pharmaceutical manufacturing is based on mass production models, considering the cost-effectiveness. As personalized medicine grows and the need for individualized treatments becomes more real, there is going to be a critical time point where changes in the manufacturing technologies of pharmaceutical industries will be essential [5]. A production method such as mass customization by modular design of pharmaceutical products has been proposed in order to achieve high personalization while also overcoming the technical and economic limitations associated with the status quo of production [57]. Although 3D printing has been induced in the pharmaceutical industries since the marketing authorization of the first 3D-printed drug product (Spritam^®^), the implementation of 3D printing is still not feasible due to the lack of suitable equipment. For example, printing on a conveyor and using successive print heads has been suggested in order to minimize the time required for the production of tablets and to eliminate the need to remove objects from the platform after printing [14]. 

**Figure 5:**
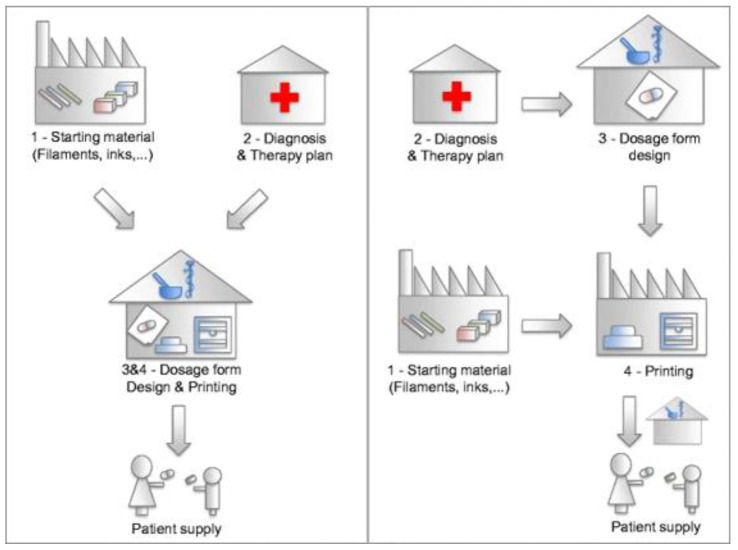
Two possible scenarios for the production of 3D-printed medicine: in the community pharmacy (**left**) or in the pharmaceutical industry (**right**). Both cases comprise an integral network between the proposed therapeutic plan, the design of a personalized dosage form, the utilization of the appropriate feedstock, and the production and final distribution of the medicinal product to the patient [55].

In the scenario of 3D printing in the pharmaceutical industry (Figure 5), the individualized medication is produced in the industry and is then distributed to the patient either directly or through intermediaries. This requires the establishment of good distribution practices (GDP) for the safe and traceable delivery to the patient [54]. It has also been mentioned that industries might be indifferent towards personalized preparations unless it were profitable and suitable to the already existing business and supply model, whereas smaller companies might express a greater interest in the implementation of this concept. Other concerns raised in this possible setting include the regulatory standards that the industry will have to meet in order to sell this type of product, the direct access of the industry to patients’ health records, as well as the direct contact of the industry with patients, something that is prohibited in the present [53]. Finally, there is also the concern that if 3D-printed medicines are produced en masse in the pharmaceutical industry, the concept of individualized on-demand manufacturing might be overlooked [55].

### 3.3. Community Pharmacy

A community pharmacy is considered a suitable setting for the application of 3D printing in medication production. The staff is well educated, patients are already accustomed to receiving their prescriptions from them, and the possibility of preparing on-demand dosage forms through compounding is a well-established procedure in most countries worldwide [56]. In the current state of the healthcare system, shortages of drugs for extended periods of time are becoming more and more common, and 3D printing could provide a solution to this problem, as in such cases, the deficient products could be manufactured directly at the point of care, i.e., the pharmacy [5]. Two different cases have been presented: in the first, both the design and the manufacturing of the medication occurs in the pharmacy, whereas in the second, the design and distribution of the product occurs in the pharmacy, and the 3D printing process takes place in a specialized facility (Figure 5) [55]. Nevertheless, pharmacies are expected to play a vital role in all cases, and this seems to be the most logical choice [53]. Since this scenario is mostly based on the current distribution model of drugs, it also seems to be more plausible. Furthermore, in this setting, pharmacies resemble more the previous-generation pharmacies, where medicine was mainly prepared on site and for a certain individual [54].

Naturally, issues have also been raised concerning this setting. For instance, there is a debate as to whether the printing of drugs in pharmacies should be optional or compulsory for all pharmacies, giving a political aspect to the discussion [56]. Additionally, questioning the revision of the pharmacists’ education must be addressed, as changes will definitely need to occur in order to include training on 3D printing, digital health, and personalized medicine [54].

### 3.4. Hospital Pharmacy

3D printing of medicine in a hospital pharmacy is one of the most realistic settings proposed in the existing literature and has been extensively discussed. This scenario includes the diagnosis of the patient; the consideration of the individual’s characteristics, such as age, body condition, medical history, and genetics; the creation of a specific profile; and the treatment plan. Then, the appropriate dosage form is designed based on available data and AI software and manufactured by the 3D printer of the hospital pharmacy before being delivered to the patient [15]. Since most hospital pharmacies already have a compounding laboratory and skilled medical staff, it should be easy to introduce 3D printing as a method of medicine manufacturing for individual patients. However, it has also been noted that from a financial point of view, it might be easier to introduce the discussed method in large university hospitals instead of small hospitals. This is also supported by the fact that university hospitals usually include large compounding facilities with better equipment and deal with more patients in need of individualized therapy [53].

Apart from the study by Zheng et al. [17], where 3D printing was employed in a hospital to treat patients individually compared to manually divided tablets, two more studies on the use of drug 3D printing in hospitals have been published to date. In the first study, Goyanes et al. applied 3D printing in a hospital to personalize the treatment of four pediatric patients with maple syrup urine disease (MSUD) [58]. Two different dosage forms containing isoleucine were developed: one capsule filled manually and one 3D-printed chewable tablet with various flavors and colors. 3D-printed formulations were superior to the conventional capsules both in terms of dose accuracy and acceptability. The authors also implied that 3D printing in a hospital setting could solve common compounding problems while also elevating the quality and safety of the final product in a fast and efficient way [58]. In a second study, Öblom et al. compared the preparation of warfarin dosage forms for pediatric patients in a hospital through printing techniques with the traditional method of powder division in unit dose sachets. 3D printing and 2D printing were used to produce orodispersible films of various doses, and the stability of these formulations was confirmed over a month. The suitability of administering these dosage forms via a nasogastric tube was also evaluated so that they could be delivered to patients of various states [59].

### 3.5. General Challenges

No matter the setting of application of 3D printing in medicine production, there are some challenges mentioned in the existing literature that might hinder the realization of these theoretic concepts.

One of the major concerns is the education of the staff handling the 3D printing equipment. Will it be the pharmacist or someone more skilled in the field of digital design and 3D printing, and will the education system need to be changed to meet the requirements that arise [15,54,55]? Additionally, one of the most commonly encountered issues is the assurance of the quality of the 3D-printed product. The need for novel non-destructive techniques and process analytical technologies (PAT) to confirm the safety and quality of the medication without tampering the sample is essential [15,55]. Although 3D printing of medicines can be realized through a one-step procedure [60,61], most technologies still require post-printing processing (e.g., drying, cooling, UV curing) to enhance the product’s mechanical stability [62]. However, this extra step might lead to further quality control issues, as it places the stability of the active pharmaceutical ingredients at risk [61] and it may affect the drug release performance [63]. Sterility, stability, and contamination problems, along with environmental aspects, for example, solvent, excipient, and waste handling, are also of great importance and need to be further debated [54]. From an economical point of view, installing 3D printers in places such as hospitals, community pharmacies, or even houses still seems to be an expensive investment that most cannot afford [15]. The supply chain of drug products, the business model, and the manufacturing protocols of pharmaceutical industries might have to be reestablished, no matter which setting is optimal, to introduce 3D printing in personalized treatment [64]. Furthermore, although the progress in the development of 3D printers is major, the scientific community is still not ready to present the most suitable device for such settings, as it has to be fast, easy to operate, and cheap but with good resolution [15]. Moreover, further research is required in order to investigate the behavior of currently used excipients and drugs under the conditions and stresses imposed by 3D printing technologies, e.g., the acceptable temperature windows for processing of thermolabile substances. For this purpose, methods that assess the thermal stability of the used substances, e.g., thermogravimetric analysis (TGA), must comprise an integral part of the evaluation procedure of pharmaceutical 3D printing [65]. Another example of the prerequisite characterization methods is the determination of the flow properties of the pastes used in semisolid extrusion 3D printing, which are necessary to establish the optimal paste properties for successful 3D printing of medicines [66]. Apart from the regulatory framework for the marketing of such products that has been widely discussed and still seems to be vague, people are concerned about the liability issues that might emerge through this practice [53,64]. Finally, access to patients’ medical records, data, and privacy are of great ethical importance, and the security of these must be ascertained [53,54].

## 4. Critical Discussion

An implementation of 3D printing technologies in the pharmaceutical manufacturing setting is in accordance with the European Union (EU) and Pharmaceutical Strategy for Europe, which consists of four pillars: (a) addressing unmet medical needs; (b) supporting competitiveness, innovation, and sustainability; (c) enhancing crisis preparedness and response mechanisms, diversified and secure supply chains, and addressing medicine shortages; and (d) ensuring a strong EU voice in the world by promoting a high level of quality, efficacy, and safety standards. 3D printing of medicinal products is consistent with all four pillars of the strategy, as it addresses unmet medical needs (for example, by providing the capability of creating personalized dosage forms for rare diseases), introduces innovative digital healthcare advantages in patient treatment, addresses supply shortages, and enhances the robustness of the healthcare systems while also improving the overall quality of pharmacotherapy [67].

1. Medicine shortages pose a major challenge in hospital settings. A recent survey conducted by the European Association of Hospital Pharmacists (EAHP) revealed that 95% of hospital pharmacists reported medicine shortages as a current problem in 2019 (an increasing trend, as the corresponding percentage for 2018 was 91,8%), whereas the most common shortages refer to antimicrobial agents (63%) and oncology medicines (47%), i.e., critical medications for the treatment of patients in the hospital setting [68]. The majority of physicians (72%), nurses (62%), and other healthcare professionals (89%) also reported that shortages have detrimental effects on patient pharmacotherapy. These shortages caused significant negative effects on patient care, as they resulted in delays in care or therapy (42%), suboptimal treatment (28%), cancellation of care (27%), and increased length of stay in the hospital (18%). According to the same source, 58% of these shortages were caused by manufacturing issues and 44% by supply chain problems. 

Therefore, 3D printing could possibly offer a solution to these shortages by facilitating the in situ manufacturing of the lacking pharmaceutical formulations. Manufacturing could refer to the 3D printing of drug formulations that are completely absent from the market, printing of specific strengths of formulations that are in shortage or with production discontinued by the industry, or the printing of formulations in personalized strengths and combinations with other medications. The resolution of shortage issues could have a beneficial effect by reducing the time required by hospital pharmacists and pharmacy technicians/assistants searching for medications in shortage, allowing them to engage in other important tasks in the provision of high quality, safe, and efficacious care. Moreover, this could reduce costs in health systems, as a more costly alternatives must often be used in the case of shortages. Finally, 3D printing would improve both the efficacy of the pharmacotherapy (as it ensures that the correct treatment is administered timely) and the perception of the care provided (as 65% of patients believed that shortages had an impact on the care provided in the hospital and 71% of patients stated that they do not feel that their health problem was properly handled). Certain patient populations in which adherence to pharmacotherapy is problematic (such as psychiatric patients) could greatly benefit from the availability of the exact dosage form required, as the ingestion of a multitude of regimens (if the shortage of a certain strength requires the intake of more than one dosage form to achieve the pertinent result) is undesired. Moreover, the adherence of such populations could be undermined by frequent change between generics due to shortages; thus, the apparent uniformity of in-house 3D-printed dosage forms could also have a positive effect on such patient categories since changes in color, shape, or texture are undesired [69].

It should be further mentioned that in situ preparations of dosage forms via compounding can provide cost-saving procedures in hospital settings, especially in cases where commercially available products are significantly overpriced. A characteristic example of such practice is the preparation of chenodeoxycholic acid Leadiant (CDCA) capsules (used to treat a rare hereditary metabolic disorder, cerebrotendinous xanthomatosis (CTX)) by an Amsterdam Hospital following the multiplication of the drug’s price by the manufacturing company [70]. 3D printing technology can significantly assist these efforts by providing means of precisely manufacturing such dosage forms.

The cost-saving efficacy of pharmaceutical 3D printing is definitely related to issues such as the patents applied to certain formulations and the extent of regulatory requirements for quality assurance. It would be very difficult to establish a cost-effective 3D printing process for a limited number of patient cases if patents are in place [5,8], so the majority of such applications would possibly employ designs and techniques after the expiration of the respective patents. The economic sustainability of drug 3D printing is a multi-factorial issue, as it is related to costs that vary significantly between different countries (for example, labor costs per hour, regulatory requirements, and cost of the raw materials and devices). Thus, a cost-benefit analysis should be performed individually for every 3D-printed formulation or drug combination used in each setting.

2. Considering another possible scenario, a collaboration between different settings (i.e., industry and hospital/community pharmacies) may occur. More specifically, the industry could manufacture the feedstock for 3D-printed pharmaceuticals, e.g., filament coils or ink cartridges, ensuring that they are good manufacturing practice (GMP)-conditioned and they contain an exact quantity of any given drug per filament length or ink volume unit. These formulations could be further used by hospital or community pharmacies to manufacture individualized dosage forms by simply determining the final volume, shape, and dimensions of the formulation in order to achieve the desired drug mass and the optimum external characteristics for each individual patient. This approach could remove the engineering challenges and the extended materials science knowledge required in order to manufacture drug-loaded filament or ink suitable for 3D printing, ensuring that hospital or community pharmacists would only have to determine the drug’s final mass and the organoleptic properties of the individualized dosage forms. This scenario could be beneficial for locations such as small islands, where the only community pharmacy (or other healthcare facility) may have to wait for several days until it receives resupply of medications.

3. The regulatory framework regarding 3D printing of medicines is another aspect that has to be considered, as the designation of 3D-printed dosage forms is crucial for the course of the implementation of this technology in drug manufacturing. More specifically, if 3D-printed drugs are considered extemporaneous preparations, the use of 3D printing in hospital or community pharmacy settings can be a viable option. If the regulatory authorities consider 3D-printed dosage forms as industrial goods, the implementation of pharmaceutical 3D printing beyond the industry becomes problematic, as the produced dosage forms would have to be subjected to rigorous testing before administration. Possible solutions to these issues would be the definition of certain brackets within which formulations could be prepared after testing dosage forms with the highest and lowest drug concentration [53]. Even if 3D-printed dosage forms are considered extemporaneous preparations, regulations vary between countries [71]. In specific situations, such as in the USA, current legislation specifies that such formulations can be manufactured by traditional compounding pharmacies, or “503A” pharmacies, only if specifically prescribed by a physician for a certain patient, largely avoiding the more burdensome regulations required for drug manufacturers under the Federal Food, Drug, and Cosmetic Act (FDCA). A second category of compounding pharmacy, called an “outsourcing facility”, can produce extemporaneous preparations in bulk after complying with the stringent current good manufacturing practice (CGMP) standards [72]. In Europe, the same applies in countries such as the Netherlands, where extemporaneous preparations can be produced in bulk by certain compounding facilities that comply with strict good laboratory practice (GLP) regulations [73]. The implementation of a uniform regulatory framework regarding compounding, such as that proposed by the EU Committee of Ministers, could resolve such issues, clarifying the borders within which pharmaceutical 3D printing could be implemented [74].

It is important to consider that not all community pharmacies will have the ability to print medications (just as today, not all pharmacies compound), but a certain proportion of larger pharmacies that have already invested in compounding procedures/facilities and have dedicated staff for these activities will find it easier to incorporate 3D printing of personalized medicines within their premises. The same principle applies to hospital pharmacies, where pharmacies located inside large university hospitals are better equipped and have more spacious facilities and trained personnel than hospital pharmacies in small regional healthcare facilities, rendering the former more suitable for the adaptation of 3D printing of personalized dosage forms [53].

**Figure 6:**
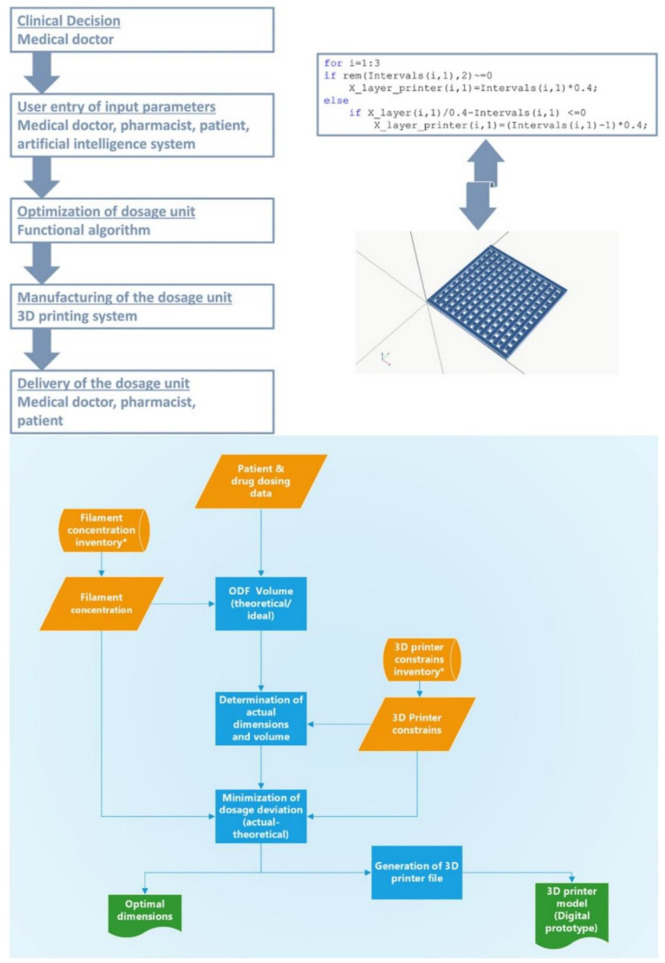
Example of a future scenario for automated digital design of customized medicinal products through an algorithm. The algorithm processes the appropriate input data, i.e., the suggested therapeutic plan and the available feedstock properties, and generates the optimal digital design of the medicinal product. Afterwards, the generated design can be loaded to the 3D printer by a medical doctor, a pharmacist, or a patient in order to proceed with manufacturing of a personalized dosage form [79].

4. Considering that current pharmaceutical manufacturing is based on a mass production model, the presence of well-established methods for the production of predetermined dosage units is apparent. However, in the case of 3D printing individualized medications in distributed points of care, there are additional issues that should be addressed, e.g., the optimal settings of the 3D printer and the inevitable step of generating the digital design of the dosage form. It should be noted that the concept of Industry 4.0 implies the utilization of automation-based models [75] and that it would be arbitrary to provide extended training on this new reality to current healthcare professionals. In order to promote automation, a previous work presented the utilization of AI in the prediction of an optimal 3D printing setup [76], whereas another work presented the processing of correlation data between the settings of the 3D printer and the incorporated drug dose, as well as its drug release performance [77]. Nonetheless, the scenario of 3D printing medicines in distributed points of care demands intercommunication platforms that guarantee the real-time access of the healthcare professional to physiological/clinical data of the patient [78]. Therefore, it would be crucial to develop specialized algorithms in the context of a simplified and user-friendly computer software that can be straightforwardly operated by healthcare professionals and process the required patient data (Figure 6). The critical level of this software would focus on automatically providing the digital design of the dosage form that is intended to be 3D-printed [79]. This would alleviate the additional burden on hospital/community pharmacists for training or the need to put in place a specialized workforce, e.g., 3D printing and digital design specialists, in the distributed manufacturing sites.

## 5. Conclusions

3D printing technology has significantly focused major research activities on promoting personalized treatment approaches. The past borders for on-demand manufacturing of pharmaceuticals in central facilities have largely expanded to real-time manufacturing at highly distributed sites, e.g., pharmacies, industries, or even houses. Critically, the option for implementing these scenarios will be realized by profoundly addressing practical issues that extend from safety-first (from the patient side), to everyday practice (from the healthcare professionals’ side). Significant changes must occur, with consideration of the current regulations and the mentality of all related professional or public parties. Nevertheless, the undeniable evidence is that 3D printing has revolutionized the way we perceive medicines, and major steps must be taken in a timely manner to realize the leap from current pharmaceutical strategies towards the pharmaceutical manufacturing concepts of the future.

## Data Availability

Not applicable.
