# Correction: Andreadis et al. The Advent of a New Era in Digital Healthcare: A Role for 3D Printing Technologies in Drug Manufacturing? *Pharmaceutics* **2022**, *14*, 609

**DOI:** 10.3390/pharmaceutics14122782

**Published:** 2022-12-13

**Authors:** Ioannis I. Andreadis, Christos I. Gioumouxouzis, Georgios K. Eleftheriadis, Dimitrios G. Fatouros

**Affiliations:** Laboratory of Pharmaceutical Technology, Department of Pharmacy, Aristotle University of Thessaloniki, 54124 Thessaloniki, Greece

## 1. Figure Legend

In the original publication [1], there was a mistake in the legend for Figure 1. The legend did not include the reproduction permission from the author. The correct legend appears below.

“Figure 1. The need for personalization of pharmaceutical products and changes in product volume and variety due to product customization according to the needs of individual patients. This figure has been reproduced with permission from © 2021 Rydvikha Govender [6]”.

## 2. Error in Figure

In the original publication, there was a mistake in Figure 1 as published. We have used the modified form of the Figure contained in a review instead of the original Figure from the creator’s PhD thesis. The corrected Figure 1, representing an unmodified version of the original figure, appears below.

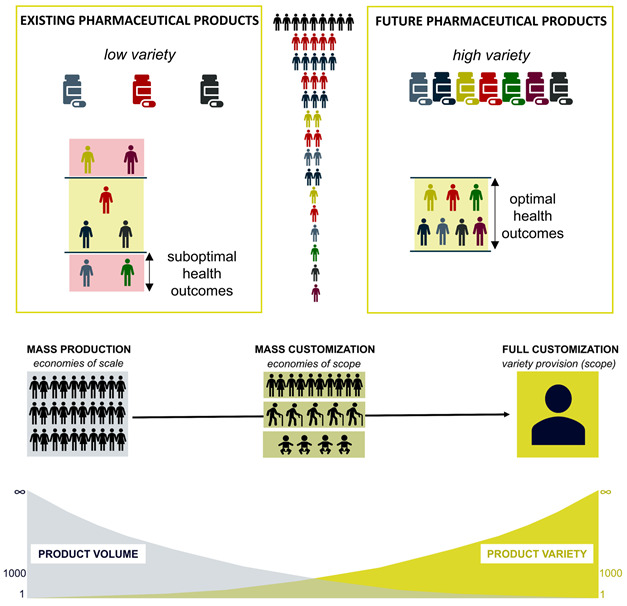



## 3. Reference

A change was made in Ref. [6] mentioned in our Figure 1, in order to provide the reference for “Rydvikha Govender, Ph.D. Thesis “Integrated Product and Process Design for Mass Customization: A Road Towards Patient Access to Individualized Pharmaceutical Therapy”, February 2021, instead of Ref. from Raijada et al. The corrected reference [6] appears below:

6. Govender, R. Integrated Product and Process Design for Mass Customization: A Road Towards Patient Access to Individualized Pharmaceutical Therapy. Ph.D. Thesis, Chalmers University of Technology, Gothenburg, Sweden, 2021.

The authors state that the scientific conclusions are unaffected. This correction was approved by the Academic Editor. The original publication has also been updated.
